# Acute liver failure caused by lymphocyte-depleted Hodgkin lymphoma in tuberculosis and HIV-infected patient

**DOI:** 10.4322/acr.2024.490

**Published:** 2024-05-22

**Authors:** Mayur Parkhi, Madhumita Premkumar, Amanjit Bal, Ashim Das, Sanjay Jain, Suvradeep Mitra

**Affiliations:** 1 Post Graduate Institute of Medical Education and Research, Department of Histopathology, Chandigarh, India; 2 Post Graduate Institute of Medical Education and Research, Department of Hepatology, Chandigarh, India; 3 Post Graduate Institute of Medical Education and Research, Department of Internal Medicine, Chandigarh, India

**Keywords:** Hodgkin Lymphoma, Liver failure, Acute, HIV, Tuberculosis

## Abstract

The lymphocyte-depleted classic Hodgkin lymphoma (LDCHL), the rarest subtype of classic Hodgkin lymphoma (CHL), is usually diagnosed at an advanced stage (stage IV) and one that unusually involves the liver, causing a rapidly progressive clinical course. We describe a 40-year-old immunocompromised man presenting with a progressive non-cholestatic jaundice and intermittent fever. The abdominal ultrasonography revealed a nodular liver with coarse echotexture and periportal hypodensities. The thoracic and abdominal contrast-enhanced computed tomography revealed right cervical and paraaortic lymphadenopathy, hepatosplenomegaly, diffuse mural thickening of duodenal and jejunal loops, and bilateral lobulated kidneys. Subsequently, he succumbed to his illness secondary to refractory septic shock. On postmortem examination, he was diagnosed with classic Hodgkin lymphoma (lymphocyte-depleted type) involving paraaortic and mediastinal lymph nodes based on morphology and immunochemistry findings. The lymphomatous process involved the liver (causing multiacinar confluent hepatic necrosis) and spleen, both showing tuberculous foci. This autopsy case depicts an uncommon case of acute liver failure due to infiltration of the liver by LDCHL in an HIV-infected patient. The findings of angiotropism and angioinvasion establish the pathological mechanism of liver failure (hepatocellular necrosis) in such cases.

## INTRODUCTION

The lymphocyte-depleted classic Hodgkin lymphoma (LDCHL), the rarest subtype of classic Hodgkin lymphoma (CHL), accounts for 1% to 1.5% of CHL among the Western population.^1^ It is considered as one end of the same spectrum with the mixed cellularity CHL because of its near similar morphological features and EBV association.^2^ Prognostically, the survival period for LDCHL is dismal compared to the remaining CHL subtypes.^3^ The CHL patients, especially the LDCHL, usually present at an advanced stage, most commonly involving the liver predominantly in the HIV status, causing rapid progressive clinical course.^4,5^ Acute liver failure as the primary manifestation in the setting of HL is occasional and usually seen in the late advanced stage of the disease. Herein, we described a 40-year-old HIV-infected patient who presented primarily with acute liver failure due to the background nodal LDCHL that was diagnosed later on postmortem histopathological examination.

## CASE REPORT

A 40-year-old male truck driver presented with progressive non-cholestatic jaundice without a prodrome, weight loss (6 kg over 1.5 months), intermittent fever (39° to 40 °C), followed by progressive abdominal distension, 2 weeks after the onset of jaundice. He had a history of a traumatic fracture of the right hip one year back, which was managed by a hip plaster cast. He also had a history of significant alcohol intake (30-40 gm/day; 5-6 times per week but was abstemious for a year). He was an occasional smoker but denied any intravenous drug use or any other addiction. He had been diagnosed with AIDS and disseminated tuberculosis seven years ago, for which he received HAART and ATT. He had also received anti-PJP (*Pneumocystis jirovecii* pneumonia) prophylaxis.

At the time of admission, his vitals were within normal limits. There was pallor, icterus, pedal edema, right cervical non-tender lymphadenopathy, and abdominal distension, and the spleen was palpable 5cm below the left costal margin. The blood parameters at admission are depicted in Table 1.

**Table 1:** The blood parameters findings of the patient at the time of admission

Parameters	Result	Normal Range
Hemoglobin (g/dl)	7	13.2-16.6
Leukocyte count (mm^3^)	3900	4000-11,000
Platelet count (x10^9^/L)	102	135-317
PT (%)	48	85-100
INR	2.07	0.8-1.1
aPTT (seconds)	56	30-40
Na (mmol/L)	136	135-145
K (mmol/L)	4	3.6-5.2
Cl (mmol/L)	96	96-106
Urea (mg/dl)	86	5-20
Creatinine (mg/dl)	1.9	0.7-1.3

PT, prothrombin time; INR, international normal index; aPTT, activated partial thromboplastin time; Na, sodium; K, potassium; Cl, chloride.

Chest X-ray was within normal limits. Ultrasonography of the abdomen revealed a nodular liver (17 cm) with coarse echotexture and periportal hypodensities. The portal vein was 18 mm at the porta, and the gallbladder was contracted. In addition, splenomegaly (19 cm), moderate ascites, and bilateral raised renal echogenicity with maintained cortico-medullary differentiation were noted. Contrast-enhanced computed tomography (CECT - chest and abdomen) revealed right cervical and paraaortic lymphadenopathy, hepatosplenomegaly, diffuse duodenal and jejunal loops thickening, and bilateral lobulated kidneys. Investigations revealed severe pancytopenia, acute kidney injury, and high-serum ascites albumin gradient, low protein ascites with a low CD4 count (17 cells/microliter). A clinical possibility of acute-on-chronic liver failure with portal hypertension in a known case of HIV/ AIDS was considered. Subsequently, he developed progressive renal failure, deteriorating sensorium, and shock with respiratory compromise over the next 4 days. He finally succumbed to his illness secondary to refractory septic shock.

## AUTOPSY FINDINGS

A complete autopsy was performed after obtaining consent from the next-to-kin. On opening the body cavities, the peritoneal cavity yielded 1.8 L of straw-colored fluid, while each pleural cavity yielded 0.3 L.

**Figure 1:**
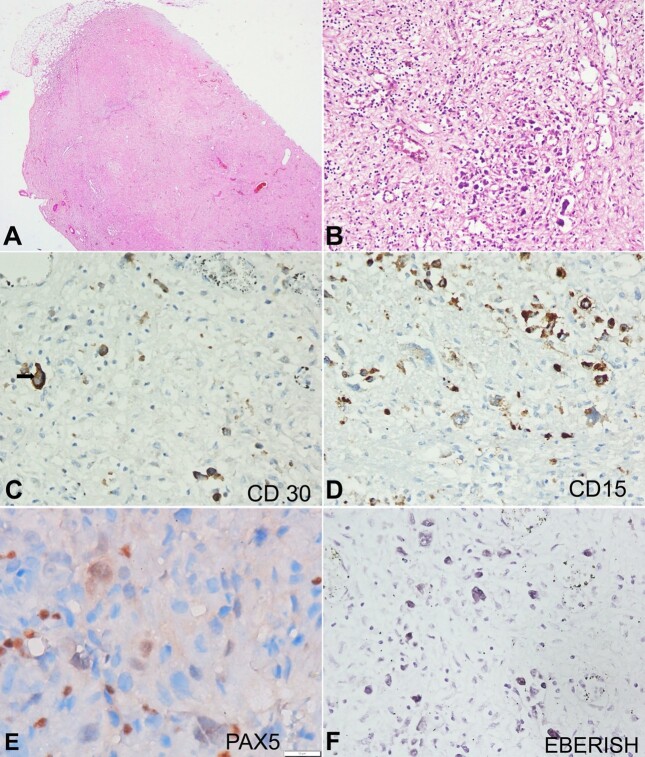
Photomicrograph of the lymph node. **A –** The paraaortic lymph nodes show diffuse effacement of the nodal architecture with few large atypical cells in a pauci-inflammatory collagenous background (H&E, 200X); **B –** These large atypical cells had pleomorphic nuclei, vesicular chromatin, macro-nucleoli, and moderate to abundant amounts of cytoplasm (H&E, 200X); **C –** CD30 showing membranous and Golgi pattern positivity (black arrow) in these atypical cells (400X); **D –** CD15 showing membranous and Golgi pattern positivity (400X); **E –** PAX5 showed dim nuclear expression (1000X, oil immersion field); **F –** EBERISH showed nuclear positivity (400X).

The lymph nodes (paraaortic and mediastinal) were enlarged (ranging from 1 to 2.5 cm in maximum dimensions), and their cut surfaces appeared homogeneous, white, and firm. These lymph nodes showed diffuse effacement of the nodal architecture with prominent collagen deposition. Interspersed were a few atypical cells in a pauci-inflammatory background (Figure 1A). These large atypical cells had pleomorphic nuclei, vesicular chromatin, macro-nucleoli, and moderate to abundant cytoplasm (Figure 1B). They were positive for CD30 (membranous and Golgi pattern) (Figure 1C) and CD15 (membranous and Golgi pattern) (Figure 1D). CD45, CD20, and CD3 were negative. PAX5 (transcription factor) showed dim nuclear expression (Figure 1E). Epstein-Barr encoding region (EBER) in situ hybridization (ISH) was strongly positive (Figure 1F). The morphology and immunohistochemistry findings pointed to the diagnosis of classic Hodgkin lymphoma, a lymphocyte-depleted (diffuse fibrosis variant).

**Figure 2:**
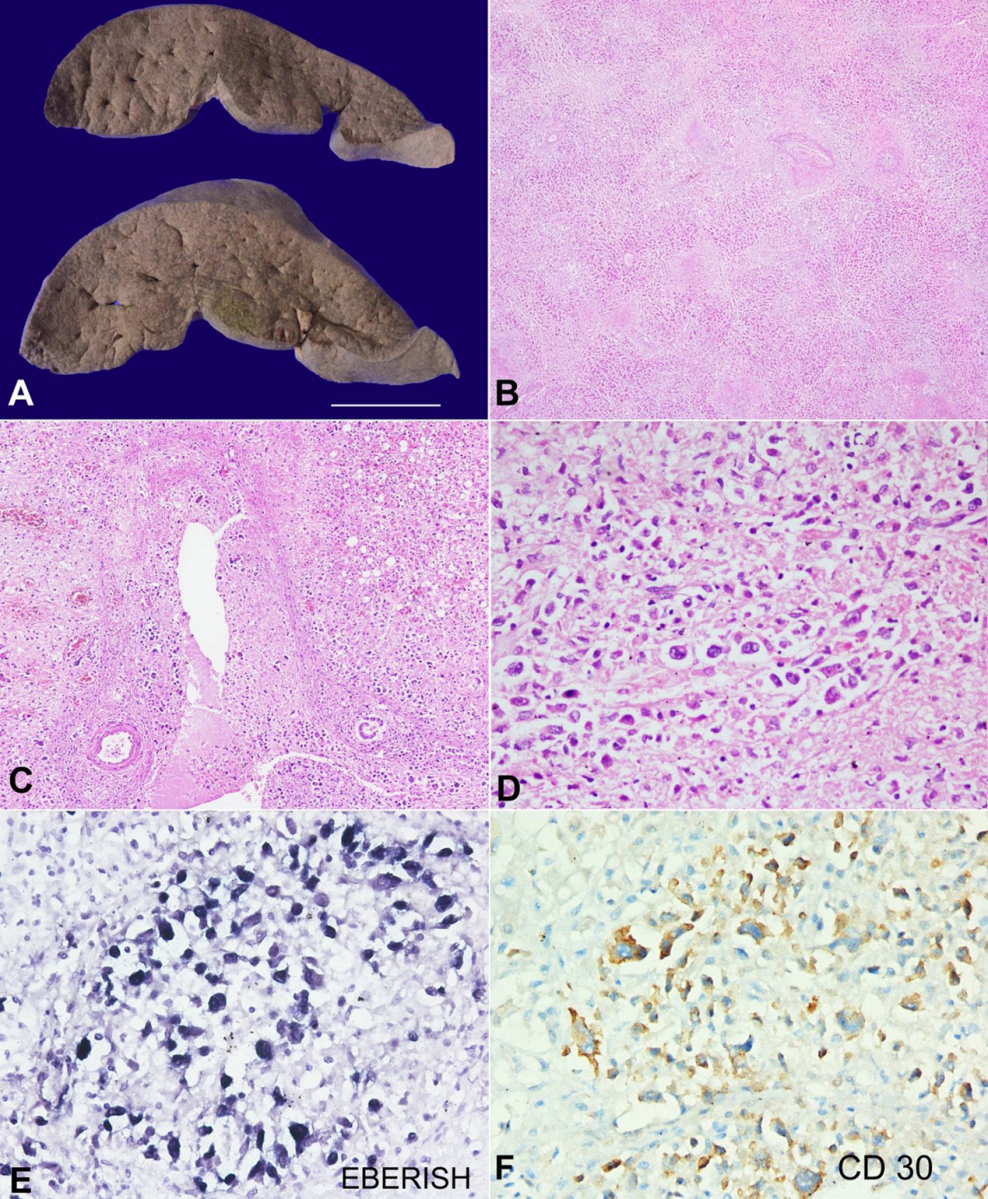
Photomicrographs of the liver. **A –** The liver was markedly enlarged with a bile-stained, mottled surface and wrinkled capsule. The liver slices were soft and foldable, although there was no nodularity to suggest background cirrhosis (scale bar = 5 cm); **B** and **C –** Microscopy showing predominantly periportal and focally panacinar multiacinar and bridging confluent hepatic necrosis due to portal phlebitis caused by infiltration of the portal venous radicles by large atypical lymphoid cells in a pauci-inflammatory background (H&E; B, 20X; C, 40X); **D –** Occasional central veins were also infiltrated by these atypical cells (H&E, 400X); **E –** EBERISH showing nuclear positivity (400X); **F –** CD30 was expressed as a membranous and Golgi pattern (400X).

The liver weighed 2550 g (RR: 1400 to 1500g) and was markedly enlarged with a bile-stained, mottled surface and wrinkled capsule. The liver slices were soft and foldable, and the cut surface revealed nodules of size 5 to 8 mm in size (Figure 2A). The portal veins, hepatic veins, and the biliary tree were patent. Microscopy showed predominantly periportal and focally panacinar multiacinar and bridging confluent hepatic necrosis due to portal phlebitis caused by infiltration of the portal venous radicles by large atypical lymphoid cells in a pauci-inflammatory background (Figures 2B and 2C). These atypical cells also infiltrated occasional central veins (Figure 2D). Hepatic arteries and the interlobular bile ducts were unremarkable. The preserved hepatocytes showed macrovesicular steatosis. These atypical cells had the abovementioned immunoprofile suggesting infiltration by LDCHL (Figures 2E and 2F). Immunohistochemistry for HBsAg, HBcAg, CMV, and p24 were negative. The portal tracts or the hepatic lobules did not show any background fibrosis.

**Figure 3:**
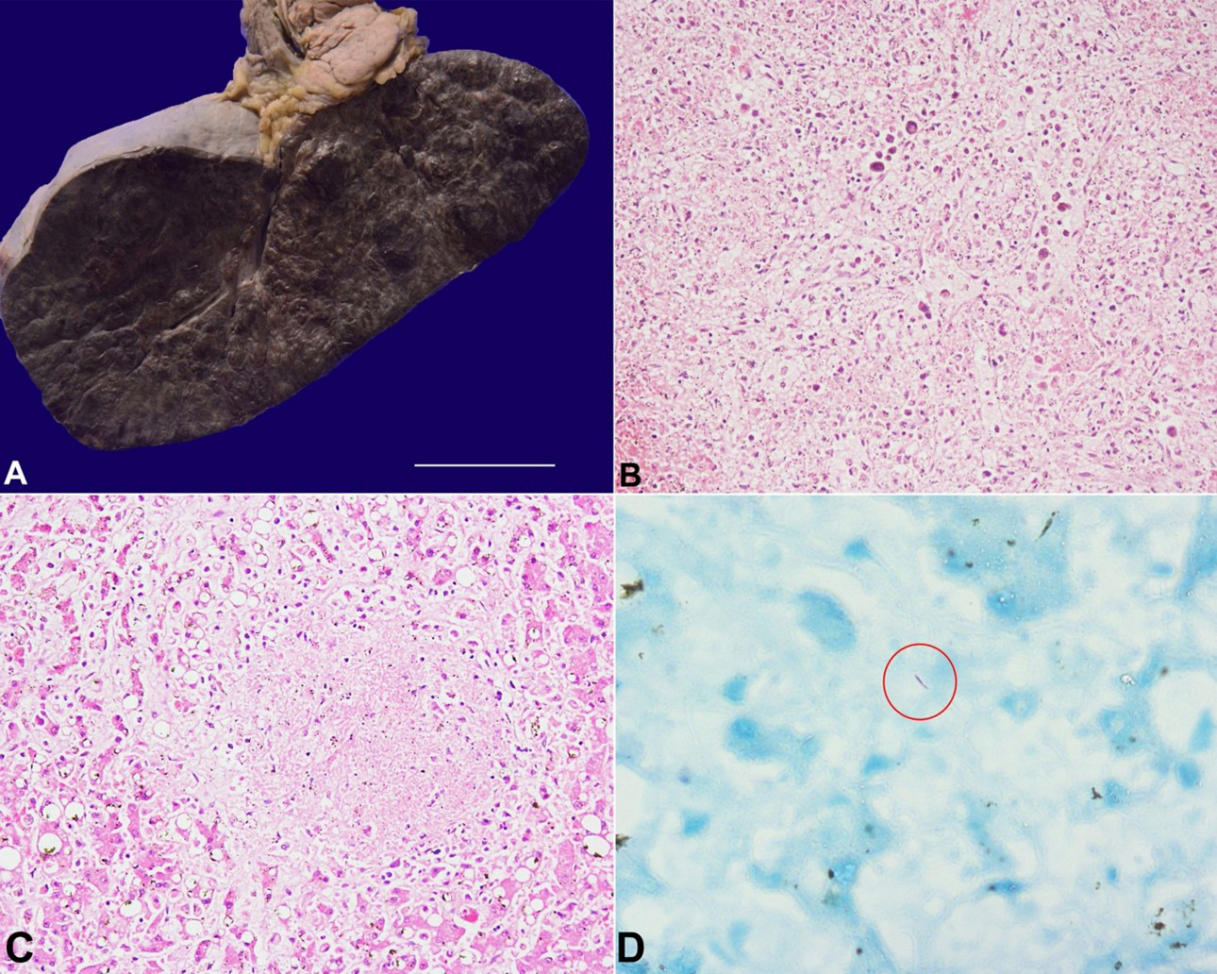
**A –** The spleen was massively enlarged with multiple elevated hemorrhagic nodules measuring 1-3.5cm (scale bar= 2 cm); **B –** These hemorrhagic nodules on microscopy showed hemorrhagic necrosis of the splenic parenchyma due to infiltration and angioinvasion by the lymphomatous cells (H&E, 100X); **C –** The liver parenchyma showed necrotizing granuloma composed of a few epithelioid cell histiocytes (H&E, 200X); **D –** Ziehl-Neelsen stain highlighting acid-fast bacillus (red round) (Oil immersion field, 1000X).

The spleen weighed 1030 g (RR: 180 to 220g) and was massively enlarged with multiple elevated hemorrhagic nodules measuring 1-3.5 cm (Figure 3A). These hemorrhagic nodules on microscopy showed hemorrhagic necrosis of the splenic parenchyma due to infiltration and angioinvasion by the lymphomatous cells (Figure 3B).

Besides, there were occasional foci of hepatic lobular and splenic parenchymal necrosis and very few epithelioid histiocytes (Figure 3C). The Ziehl-Neelsen (ZN) stain for acid-fast bacilli (AFB) was positive in these necrotic foci of both the liver and spleen (Figure 3D). Bone marrow was moderately cellular, with an increase in the number of macrophages and many hemophagocytic figures, although there was no infiltration by the lymphoma cells.

The kidneys showed benign nephrosclerotic scar, bile cast nephropathy, and nephrocalcinosis. The brain showed hypoxic changes, and the heart showed concentric left ventricular hypertrophy with atherosclerotic plaques over the aorta. All other organs showed normal morphology.

In brief, this patient had classic Hodgkin lymphoma (lymphocyte-depleted type) (Stage IV) involving paraaortic and mediastinal lymph nodes with dissemination to the liver (causing multiacinar confluent hepatic necrosis) and spleen. The lymphomatous infiltration was the reason for its presentation as a hepatic failure. Besides, he had tuberculosis (confirmed on liver and spleen tissue sections) and secondary hemophagocytosis. Other features were benign nephrosclerosis, bile cast nephropathy, hypoxic changes (brain), aortic atherosclerosis, and borderline concentric left ventricular hypertrophy.

## DISCUSSION

Classic Hodgkin lymphoma (CHL) contains a few large neoplastic cells scattered or clustered in a characteristic reactive immune microenvironment. These neoplastic cells show a defective B-cell expression program.^6^ CHL is subdivided into four subtypes: nodular sclerosis, mixed cellularity, lymphocyte rich, and lymphocyte depleted; nodular sclerosis is the most common. Morphologically, the uncommon subtype termed LDCHL shows similar large neoplastic cells but a sparse inflammatory background. Compared to the other CHL subtypes, these patients usually show more common hepatic involvement in the late course of the disease and a significantly dismal prognosis.^3,4^

The patients with LDCHL show a wide age distribution, male preference, and advanced stage presentation at the time of diagnosis.^3^ Though the etiology is unclear, genetic factors and viral infections play an important role in the pathogenesis.^7^ The index patient was in the 4^th^ decade of his life with a background history of HIV and tuberculosis. Among these patients, EBV and HIV infections are seen in 60% to 72% and 15% of cases, respectively.^3,5^ The latent membrane protein 1 (LMP-1) of EBV infection not only enhances the expression of PD-L1 on the EBV-infected B-cells through various survival pathways (JAK-STAT, NF-kβ, PI3K/AKT) but also allows it to undergo activation, proliferation, and differentiation.^8^ The interaction of the HIV infection with CD40-ligand-bearing virions and HIV-associated proteins causes chronic B-cell activation.^9^ Morphologically, LDCHL shows classical HRS cells with two morphological patterns: diffuse fibrosis and reticular variant.^7^ The diffuse fibrosis variant was seen in the index case with an abundance of HRS cells against the background of a significant amount of fibrosis and a paucity of reactive inflammatory cells.

Hepatic involvement is common, varying from mild dysfunction to fulminant hepatic failure.^10-12^ The index patient also showed hepatic manifestations as reflected clinically, but the diagnosis of HL was interpreted and made on postmortem histopathological examination. HL with hepatic dissemination also shows splenic involvement.^10^ The spectrum of liver damage seen in HL includes hepatitis, liver infiltration, biliary obstruction, sepsis, vanishing bile duct syndrome, hemophagocytic lymphohistiocytosis (HLH), posttransplant lymphoproliferative disorders, liver adverse effects of chemotherapy or peliosis hepatis.^12,13^ Thorough sampling of the postmortem liver specimen revealed large neoplastic cells invading the portal venous radicles, causing periportal multiacinar and bridging confluent hepatic necrosis. In addition, microscopic necrotic foci within the liver parenchyma represented mycobacterial tuberculosis. HL and TB can coexist together but with a rare incidence rate.^14^ This association may sometimes be a nightmare as it may delay diagnosing each other.^15-17^ In HL, the suppression of cell-mediated immunity may play a role in concomitant reactivation or infection of tuberculosis.^14,15^ To the best of our knowledge, we could not find an association between lymphocyte-depleted Hodgkin lymphoma and tuberculosis, especially in the liver and spleen. This association is a rare incidence, and sometimes, During the diagnosis of tuberculosis on fine needle aspiration of the necrotic lymph node and bone marrow aspirate material, no atypical or HRS-like cells were seen or mentioned in the report. The postmortem excised lymph nodes showed the classic HRS cells against the background of abundant collagen material and pauci inflammatory cells. Secondary HLH can occur in the setting of the lymphomatous process and immunosuppressed status, both of which were evident in our patient.^18,19^ These conditions usually trigger HLH by uncontrolled release of the pro-inflammatory cytokines that cause multi-organ failures which ultimately lead to death. The deceased patient must have undergone a similar sequence of events.

## CONCLUSION

Acute liver failure (ALF) in an adult is usually caused by infection by hepatotropic/ non-hepatotropic viruses and/ or drugs/ toxins. Infiltration by a lymphoma, especially Hodgkin lymphoma presenting as ALF, is an unusual condition. This autopsy case depicts an uncommon case of ALF due to infiltration of the liver by LDCHL in an HIV/ AIDS individual. Besides, this case depicts the angiotropism and angioinvasion by the lymphoma cells and thus establishes the pathological mechanism of liver failure (hepatocellular necrosis) in these cases. These features, along with the coexistence of tuberculosis, should be considered in explaining liver dysfunction in an HIV-infected individual.
